# A review on the accuracy of bladder cancer detection methods

**DOI:** 10.7150/jca.28989

**Published:** 2019-07-08

**Authors:** Chao-Zhe Zhu, Hua-Nong Ting, Kwan-Hoong Ng, Teng-Aik Ong

**Affiliations:** 1 Department of Biomedical Engineering, Faculty of Engineering, University of Malaya, Kuala Lumpur, Malaysia; 2 Department of Biomedical Imaging, Faculty of Medicine, University of Malaya, Kuala Lumpur, Malaysia; 3 Department of Surgery, Faculty of Medicine, University of Malaya, Kuala Lumpur, Malaysia

**Keywords:** Bladder cancer, Methods, Sensitivity, Specificity.

## Abstract

**Background and purpose**: Bladder cancer is the most common malignant tumour in the urinary system, with a high incidence and recurrence rate. While the incidence of bladder cancer has been rising in recent years, the prevalence of bladder carcinoma is showing an increasing tendency in the younger age group. There are several methods to detect bladder cancer, but different methods have varying degrees of accuracy which intrinsically depends on the method's sensitivity and specificity. Our aim was to comprehensively summarize the current detection methods for bladder cancer based on the available literature, and at the same time, to find the best combination of different effective methods which can produce a high degree of accuracy in detecting the presence of cancerous cells in the bladder.

**Materials and Methods**: We used key word retrieval method for searching related references in English that had been indexed in PubMed and Medline.

**Results and Discussion**: This paper discussed the different detection methods and their sensitivities/specificities as well as the advantages and disadvantages. We summarized the best identified cancer cell detection methods with higher sensitivity/specificity.

**Conclusion**: The results of this review can positively help to identify accurate methods for detecting bladder cancer and highlight areas to be further improved for future research work.

## 1. Background

Bladder cancer is the sixth most common disease in men and the seventeenth most common in women 1. Its incidence ranks first among malignant cancers of the urinary system and second only to prostate cancer in Western countries. The pathologic histology shows that more than 90% bladder cancer patients have bladder transitional cell carcinoma, 5% have bladder squamous cell carcinoma, and less than 2% have bladder adenocarcinoma 2. Moreover, the incidence of bladder cancer is three to four times higher in men than in women 3, 4. Among patients who receive an initial diagnosis of bladder cancer, 70% to 85% have non-muscle-invasive bladder cancer (NMIBC) and 15% to 30% have muscle-invasive bladder cancer (MIBC) 5. NMIBC is known as superficial bladder cancer; its pathological stages include Ta (papillary), T1 (infiltration lamina propria), and carcinoma in situ. Ta patients comprise 70% of cases, T1 roughly 20% and carcinoma in situ about 10%. MIBC is known as invasive bladder cancer; its pathological stages include T2, T3 and T4 5, 6. Up to 80% of NMIBC patients relapse within 5 years; 30% of Ta patients progress to MIBC; while those with T1 and carcinoma in situ are more likely to develop MIBC 7, 8. Transurethral resection is seen as a standard curative treatment for NMIBC, whilst radical cystectomy plus neoadjuvant chemotherapy is used to treat MIBC 5, 9.

The pathogenesis of bladder cancer is complex and multifactorial due to either intrinsic genetic factors or external environmental factors. Two major confirmed factors are smoking and prolonged exposure to aromatic amines. Smoking is the most confirmed pathogenic factor, as about 30% to 50% of bladder cancers can be ascribed to smoking, which can amplify the incidence of bladder cancer by two to four times. The incidence rate is in proportion to the intensity and duration of smoking. Haematuria is the earliest and most common symptom of primary bladder cancer. The nature of haematuria includes full-course, intermittent and painless gross haematuria, sometimes accompanied by blood clots 8, 10. Other clinical manifestations at the initial diagnosis include microscopic haematuria, lower urinary tract symptoms and urinary tract infection 11. Statistics show that bladder cancer has high incidence, progression and recurrence rates. Therefore, in clinical work it is extremely important to accurately diagnose and assess patients with early bladder cancer and especially to monitor high-risk postoperative bladder cancer patients. The most common ways to diagnose bladder cancer include cystoscopy and biopsy, imaging methods, urinary cytology, fluorescence in situ hybridization, and urine protein detection (BTA-STAT, BTA-TRAK, NMP22 and ACCU-DX) 8, 12. Urinary cytology and cystoscopy/biopsy are the current gold standard examination tools to diagnose bladder cancer. However, cystoscopy is an invasive examination and can cause pain, bleeding, urinary tract infections and other complications. In addition, it is sometimes difficult for cystoscopy to detect tumours in secluded corners of the bladder, which constrains its clinical application. Cytology is a non-invasive test that can directly identify tumour cells shed in the urine. It is simple to use and inexpensive and performs well, although it has low sensitivity and low diagnostic efficiency, especially with low-grade bladder cancer.

## 2. Materials and Methods

**Figure 1:**
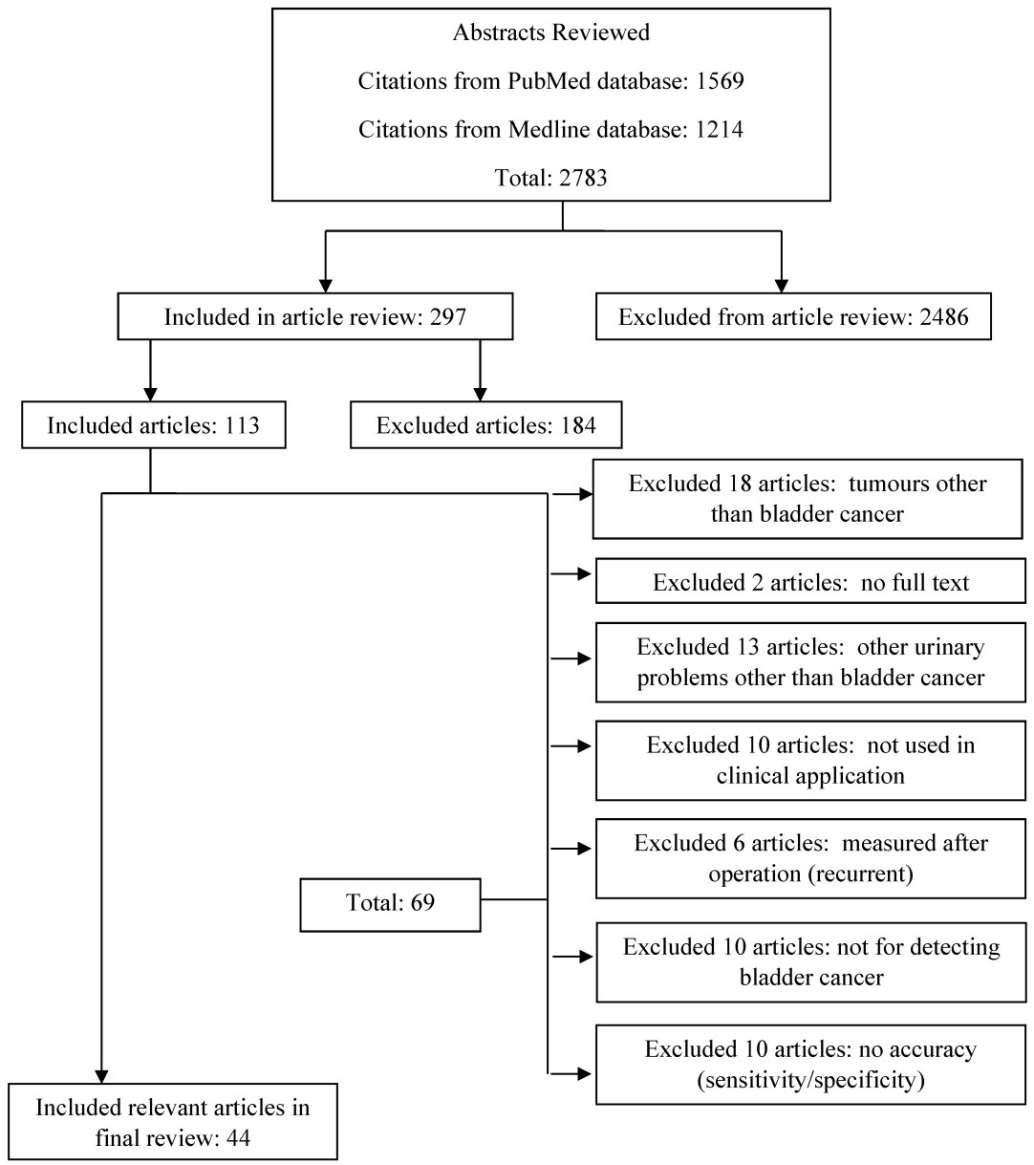
Flowchart of literature search results using Medline complete and PubMed from 2008 to 2018.

This study used the keyword information retrieval method. References related to bladder cancer detection were collected, summarized and organized to select representative and reliable research articles that matched the study's requirements. PubMed and Medline complete were used for systematic retrieval from 2008 to 2018. First, we set two keywords ('bladder cancer' and 'detection') for abstract retrieval, resulting in 2783 relevant English-language papers. Second, 297 full texts met the inclusion criteria by including the results of detection of bladder cancer with or without a clinical trial. We then used 'bladder cancer, detection' as abstracts and 'clinical trial, accuracy' as full articles that had been reviewed. After all exclusions, 44 relevant articles were obtained. The main exclusion criterion was that the tests had not been used for clinical diagnosis. Other publications regarding urinary problems and other cancers were also excluded. The flowchart for the entire article search is presented in Figure 1.

All diagnostic methods for clinical use have their own pros and cons; therefore, it becomes a top priority to find a method of detection and diagnosis of bladder cancer that is characterized by high sensitivity, high specificity, low cost, non-invasive nature, ease of use and good reproducibility. Sensitivity and specificity are both widely used to portray the results of diagnoses in the medical diagnosis field. Specificity examines healthy subjects who show negative results, and sensitivity examines those with cancer who show true positive results. Therefore, the use of sensitivity and specificity to compare several diagnostic methods can provide more reference information for the patients. In this study, we compared the diagnostic accuracy of various methods, so any article without information regarding such measures was excluded. In the end, we used 21 articles from PubMed and 23 articles from Medline complete for analysis.

## 3. Results and Discussion

This section summarizes the bladder cancer detection methods, their sensitivities and specificities and their advantages and disadvantages.

### 3.1 Urine microscopy

Urine microscopy serves as a crucial tool to determine several conditions that can affect the kidneys and the urinary tract. Nephrologists and pathologists are both responsible for microscopic examination of urine 13. Urine samples should be acquired for microscopy in the relevant examining centre using an aseptic method. Patients have no risk of injury or bacterial infection (unless urine is collected via catheterization). However, this method also has some disadvantages. The sample may include debris such as bacteria and exudates from the lower urinary tract or genital tract. If there is an increase in bacteria in the urine, it is necessary to properly identify whether its origin is urethral pollution or urinary tract infection. Catheterization performed to collect urine may also induce trauma to the urinary tract 14. Table 1 shows a review of the sensitivity and specificity of urine microscopy.

### 3.2 Urine cytology

Urinary cytology is performed under a microscope to screen the urine of a patient with bladder cancer for cancer cells. If the sample includes abnormal cells, the doctor will ask the patient for another sample. Table 2 shows a review of the sensitivity and specificity of urine cytology. Based on the review, Table 3 shows the advantages and disadvantages of the detection method.

### 3.3 Urine markers

Urine markers are a combined method for bladder cancer diagnosis. Although more than 30 urinary biomarkers are recognized to diagnose bladder disease, only a few can be used 23. It is universally acknowledged that bladder cancer should be diagnosed with urine tests such as the UroVysion test, ImmunoCyt test or NMP-22 test. Each of these examinations is conducted by recognizing chemicals, proteins and changes in chromosomes in the urine 24. Commercially available tests include:

- Urine cytology
- Fluorescence in situ hybridization (FISH)
- Nuclear matrix protein (NMP-22)
- BTA *stat*
- BTA TRAK
- ImmunoCyt/uCyt+
- CertNDx
- CxBladder

When surveying a marker's operation, researchers should consider the research population because it could have an influence on the operation of a marker at a later stage of the illness. Although the mark of the machine may be low, several markers have been subjected to focused research. Based on the review of urine markers and their sensitivity and specificity given in Table 4, it can be seen that the advantages of this method include its high sensitivity, greater number of choices, less pain and detection of low-grade tumours. In contrast, its disadvantages mainly include its high cost, invasive nature, complexity and high inter-observer variability 25.

### 3.4 Cystoscopy

This is the most vital test for diagnosing bladder cancer using cystoscope. Patients can undergo this test under local anaesthesia or general sedation. If tissue sampling is required, cystoscopy must take place under general anaesthesia 34. Based on the review of the method and its sensitivity and specificity in Table 5, it can be seen that one advantage is that it is easily performed 35. Every alternative method should be examined and evaluated with the doctor, who can confirm which choice will work best for the patients. However, this method has disadvantages; it might miss a small flat tumour, and it involves instrumentation and thus a risk of urethral injury, urinary tract infection and haematuria 36.

### 3.5 CT

A computed tomography (CT) is not used for screening but is used for staging once bladder cancer is diagnosed via cystoscopy and biopsy or via transurethral resection. A CT urogram is an imaging test to examine the urethra system that uses X- rays to generate multiple images of a part of a subject's body (e.g., blood vessels, soft tissue); these images are then sent to a computer for reconstruction of detailed three-dimensional images 41. The advantages of this method include: (1) it completely eliminates the superimposition of images of structures outside the area of interest; and (2) the statistics gained from one CT image, which comprises multiple contiguous or one helical scan, are considered images in the axial, coronal or sagittal planes, relying on the diagnostic task, which is seen as multi-planar reformatted imaging. The disadvantages of this method include: (1) a slight increase in the risk of cancer in later life via exposure to ionizing radiation (X-rays); (2) its use of higher doses of radiation than normal X-ray scanning makes the risks (while still small) greater than other types in general; and (3) injection of a contrast medium (dye) can lead to kidney insult or allergic reaction 42. Table 6 shows a review of the CT scan method and its sensitivity and specificity.

### 3.6 MR imaging

Bladder cancer is staged mainly with magnetic resonance imaging (MRI), a form of tomography that uses a powerful magnetic resonance phenomenon to extract electromagnetic signals from the body and reconstruct body information 47. Before starting the MRI scan, it is essential that any metal articles (e.g., watches, jewellery, piercings, etc.) be removed from the subject's body because metal can affect the image quality and hence alter the diagnosis 47. Because no radiation process is involved in MRI, it presents no danger to individuals who are required to avoid radiation, such as pregnant women or children, and it is easy to perform. Scanning is carried out in an enclosed space, so individuals who are claustrophobic or are otherwise unable or unwilling to remain in an enclosed space may have trouble undergoing MRI. MRI scanners often makes significant noises and require large amounts of electric current, and their cost is usually high 48. Table 7 shows a review of the MRI method and its sensitivity and specificity.

### 3.7 Combined method - Urine Markers and Urine Cytology

A combined method diagnosis system combines one or more methods. This combination will increase the accuracy of detecting cancer or other problems in the human body. The combined use of urine markers and urine cytology can improve bladder cancer detection. Table 8 compares the sensitivity and specificity of various combined urine markers and urine cytology methods.

All in all, for the selection of the bladder tumour marker to be economically affordable, the medical equipment must check the bladder pathological area completely 58. Screening and examination methods that are powerful and specific should be made immediately and completely available to doctors. However, urine cytology is sometimes affected by lower efficiency with voided quality grades 39.

## 4. Conclusion

The methods considered in this study clearly have their respective strengths. The sensitivity and specificity data in the articles were collected from various patients. We can understand the higher detection performance with the methods used. The best accuracy can also be compared between the single and combined methods. Table 9 shows the methods and the percentage range of sensitivity and specificity for each method. It can be concluded that the methods for detecting bladder cancer can be used with different modes of varying characteristics. Some of the methods are highly accurate but some are not. Nevertheless, each method has its own advantages and disadvantages.

Table 9 shows that urine makers and cystoscopy have the same highest sensitivity and the highest specificity of 97.2% and 97%, respectively. The table also shows that of the combined methods, the highest sensitivity of 94% and the highest specificity of 90% are found in urine markers and urine cytology. Bladder growth is caused by a combination of cancer-causing agents and might have a variable history. Although superficial bladder tumours occur more often, they have a tendency to progress. Each examination should ensure proper diagnosis, arrangement and evaluation.

## Figures and Tables

**Table 1:** Sensitivity and specificity of urine microscopy for bladder cancer

Authors	Year	Sensitivity/specificity (%)	References
Nataraju et al.	2018	87 / 92	15
Becker et al.	2016	87.5 / NA	16
Williams et al.	2010	91 / 96	17

**Table 2:** Sensitivity and specificity of urine cytology for bladder cancer

Authors	Year	Sensitivity/specificity (%)	References
Kumar et al.	2017	13.3 / 100	18
Lee et al.	2015	86 / 73	19
Anai et al.	2014	83 / NA	20
Hajdinjak	2008	42 / 96	21

**Table 3:** Advantages and disadvantages of urine cytology 22

Specimen Type	Advantages	Disadvantages
Voided urine	Non-invasive No instrumentation artifact	Low cellularity Vaginal contamination Poor preservation
Catheterized	High cellularity	Invasive Instrumentation artifact Poor preservation
Bladder washing	High cellularity Good cell preservation	Invasive Instrumentation artifact
Upper tract washing	High cellularity Good preservation Selective sampling	Invasive Instrumentation artifact
Brush cytology	Selective sampling	Invasive Air drying possible (if direct smear)

**Table 4:** Sensitivity and specificity of urine markers for bladder cancer

Urine markers	Sensitivity/specificity (%)	References
BTA TRAK	72-99 / 12.1-78	26-28
BTA stat	56-83 / 72-85.7	27, 29
NMP22	51-100 / 73-90	27, 28
UroVysion	80-86 / 61-86	21, 30
ImmunoCyt	68.1-72.5 / 65.7-72.3	31, 32
UBC	53.8 / 97.2	26
TMPRSS2:ERGFusion	45.4 / 34.8	33

Abbreviation: UBC=urinary bladder cancer antigen

**Table 5:** Sensitivity and specificity of cystoscopy for bladder cancer

Authors	Year	Method	Sensitivity/specificity (%)	References
Ciudin et al.	2015	Air cystoscopy	88 / 97	36
Horstmann et al.	2014	PDD cystoscopy	92 / 57	37
Shen et al.	2012	NBI or WLI cystoscopy	87.8 / 77.1 (NBI) 68.3 / 82.9 (WLI)	38
van Rhijn et al.	2009	Urethra-cystoscopy	75 / 83	39
Allam et al.	2009	Cystoscopy	100 / 94.4	40

Abbreviation: PDD=photodynamic diagnosis; NBI=narrow band imaging; WLI=white light imaging

**Table 6:** Sensitivity and specificity of CT scan for bladder cancer

Authors	Year	Method	Sensitivity/specificity (%)	References
Lu et al.	2012	CT for staging or restaging	82 /89	43
Harkirat et al.	2010	CT for restaging CT for restaging	53.8 / 77.8 86.7 / 100	44
Swinnen et al.	2010	CT for staging CT for staging	46 / 97 46 / 92	45
Sadow et al.	2008	CT for detection	79 /94	46

**Table 7:** Sensitivity and specificity of MRI for bladder cancer

Authors	Year	Method	Sensitivity/ specificity (%)	References
Lee M et al.	2017	MRI for staging	80.8 / 77.8	49
Daneshmand et al.	2012	DGE-MRI for staging	87.5 / 91.5	50
Rajesh et al.	2011	MRI for staging	78.2 / 93.3	51
Watanabe et al.	2009	MRI for staging	70 / 79	52

Abbreviation: DGE=dynamic glucose-enhanced

**Table 8:** Sensitivity and Specificity of urinary markers combined urine cytology

Method	Sensitivity/ Specificity (%)	References
NMP 22 + Cytology	73-94 / 84-90	53, 54
BTA Stat + Cytology	91-93 / 78-90	53, 54
BTA TRAK + Cytology	68-72 / 53-75	53, 55
FDP + Cytology	68-89 / 50-78	53, 56
ImmunoCyt + Cytology	72.8-90 / 64.4-78	31, 32, 54
UroVysion FISH + Cytology	61.9-72 / 83-89.7	21, 57

Abbreviation: FDP=fibrinogen degradation products

**Table 9:** The percentage range of sensitivity and specificity in different detection methods

Methods	Sensitivity/Specificity (%)
Urine microscopy	87-91 / 92-96
Urine cytology	13.3-86 / 73-100
Urine markers	45.4-100 / 12.1-97.2
Cystoscopy	68.3-100 / 57-97
CT	46-86.7 / 77.8-100
MRI	78.2-87.5 / 77.8-93.3
Urine Markers + Urine Cytology	61.9-94 / 50-90
